# Three-Dimensional Printed Filters Based on Poly(ethylene glycol) Diacrylate Hydrogels Doped with Silver Nanoparticles for Removing Hg(II) Ions from Water

**DOI:** 10.3390/polym16081034

**Published:** 2024-04-10

**Authors:** Luca Burratti, Federica Bertelà, Michele Sisani, Irene Di Guida, Chiara Battocchio, Giovanna Iucci, Paolo Prosposito, Iole Venditti

**Affiliations:** 1 Department of Sciences, Roma Tre University of Rome, Via della Vasca Navale 79, 00146 Rome, Italy; federica.bertela@uniroma3.it (F.B.); chiara.battocchio@uniroma3.it (C.B.); giovanna.iucci@uniroma3.it (G.I.); iole.venditti@uniroma3.it (I.V.); 2 Prolabin & Tefarm S.r.l., 06134 Perugia, Italy; michele.sisani@prolabintefarm.com (M.S.); irene.diguida@prolabintefarm.com (I.D.G.); 3 Department of Industrial Engineering, University of Rome Tor Vergata, Via del Politecnico 1, 00133 Rome, Italy; paolo.prosposito@uniroma2.it

**Keywords:** silver nanoparticles, surface plasmon resonance, poly(ethylene glycol) diacrylate hydrogel, 3D printing, water remediation, heavy metal ions filtering

## Abstract

Nowadays, due to water pollution, more and more living beings are exposed to dangerous compounds, which can lead to them contracting diseases. The removal of contaminants (including heavy metals) from water is, therefore, a necessary aspect to guarantee the well-being of living beings. Among the most used techniques, the employment of adsorbent materials is certainly advantageous, as they are easy to synthesize and are cheap. In this work, poly(ethylene glycol) diacrylate (PEGDA) hydrogels doped with silver nanoparticles (AgNPs) for removing Hg(II) ions from water are presented. AgNPs were embedded in PEGDA-based matrices by using a photo-polymerizable solution. By exploiting a custom-made 3D printer, the filters were synthesized. The kinetics of interaction was studied, revealing that the adsorption equilibrium is achieved in 8 h. Subsequently, the adsorption isotherms of PEGDA doped with AgNPs towards Hg(II) ions were studied at different temperatures (4 °C, 25 °C, and 50 °C). In all cases, the best isotherm model was the Langmuir one (revealing that the chemisorption is the driving process and the most favorable one), with maximum adsorption capacities equal to 0.55, 0.57, and 0.61 mg/g, respectively. Finally, the removal efficiency was evaluated for the three temperatures, obtaining for 4 °C, 25 °C, and 50 °C the values 94%, 94%, and 86%, respectively.

## 1. Introduction

Water is the most crucial good for life-support processes in organisms, and it is a basic and indispensable need for human beings and all living creatures on earth. The consumption of water by humans should be safe, easily accessible, adequate, and free from any kind of contamination. Pollutants in water pose a severe threat to human health as well as the aquatic ecosystem. Water pollutants sources are commonly split into two groups: natural and anthropogenic [1]. The natural sources refer to contamination due to geological composition or to natural events such as volcanic eruptions or geothermal fluids that change the chemical composition of water bodies. Meanwhile, anthropogenic pollution is attributed to all human activities, such as the widespread use of pesticides and manures on farms, dumping without treatments of industrial waste directly in rivers or sea, etc. [2].

Pollutants can be classified on the basis of their chemical nature, such as inorganic [3,4,5,6] and organic contaminants [7,8,9,10], pathogens [11,12,13], thermal pollution [14,15], and radioactive contamination [16,17,18]. Among inorganic contaminants, heavy metal ions represent the most dangerous species, as they are highly toxic even at very low concentrations. Moreover, they are not degradable and tend to accumulate, consequently causing damage to the environment and living beings [19]. For example, mercury (Hydrargyrum, Hg) is considered a heavy metal and is toxic at even low exposures [20]. The history of mercury compounds and their use is ancient. Indeed, the Hg amalgam cinnabar was applied in ancient cave paintings for red colors, and metallic Hg was recognized in early Greece where it was applied as a beauty product to improve skin color [21]. Inorganic mercury is transformed into organic compounds, like methylmercury (CH_3_Hg), which is extremely stable and accumulates in the food chain. CH_3_Hg is a neurotoxic compound, which is accountable for microtubule damage, mitochondrial harm, lipid peroxidation, and the gathering of neurotoxic molecules, such as serotonin, aspartate, and glutamate [22].

For these reasons, the removal of water contaminants is a topic that in recent decades has become increasingly studied and deeply explored by the scientific community. Indeed, the number of peer-reviewed papers related to this subject has grown from 17,524 to 44,518 in only nine years (2013–2022), according to the SciVal website [23], by using the keywords “wastewater treatment”, “wastewater”, and “water management”.

The techniques able to remove these pollutants include physical, chemical, and biological methods. Physical processes include sedimentation, degasification, and filtration [24,25]. Chemical approaches provide flocculation/coagulation, ozonation, chemical precipitation, adsorption, and ion exchange [26,27,28,29,30,31]. Aerobic and anaerobic treatments and phytoremediation belong to biological methods [32,33,34,35,36,37]. Physical methods, such as sedimentation or filtration, are generally used to remove particles suspended in water and, therefore, the efficiency of these techniques is limited to the size of the species to be removed. Consequently, well-solubilized chemical compounds are not affected by these techniques [38,39]. The degasification acts only on solubilized gasses, and the efficiency decreases when the gasses concentration is high [40]. Flocculation/coagulation may not be effective on species such as heavy metals or pathogens; the addition of coagulation chemicals can leak into the environment, polluting it [41]. The ozonation technique, in addition to being expensive, is ineffective for removing heavy metals or persistent organic compounds [42,43]. Chemical-precipitation efficiency depends on temperature and pH [44]. Instead, the ion-exchange method is not able to remove organic species and the membranes used reach saturation and, therefore, lose effectiveness over time [5]. Biological methods have as disadvantages the high initial cost to build the water treatment plant and the very long time of water depuration [40,45]. These are only some of the disadvantages of the purification processes mentioned.

Among all these processes, the adsorption technique has several advantages: low operational cost, relatively high efficiency, design simplicity, and in most cases, possible regenerative procedures. However, adsorption also has a disadvantage related to poor selectivity and disposal issues [40]. Nanomaterials, which have interesting features, such as optical [46,47,48], mechanical [49,50,51], electrical [52,53,54,55], or magnetic properties [56,57,58], differ considerably when compared with their macroscopic counterparts, but the most useful property is the high surface area, which makes the nanomaterials suitable as adsorbent species. Indeed, a greater surface area guarantees greater interaction with the surrounding environment. These innovative materials can be used alone or in combination with a matrix that can host them and maintain their adsorption capacity. Surface-functionalized nanoparticles (based on metal or metal oxide) are successfully used to collect heavy metal ions [59], but in this case, the main challenge is represented by the separation of nanomaterials from wastewater. For this reason, many researchers turn to designing substrate-based adsorbent materials. Indeed, with the help of polymer, it is possible to handle and collect in an easy way the sorbent filter from the wastewater, once they have removed the toxic contaminants. Recently, adsorbent materials containing noble metal nanomaterials [60,61,62,63] have been achieving success not only for their efficiency but also for other features such as anti-bactericity [64,65].

In this work, silver nanoparticles were employed as dopants in three-dimensional filters based on PEGDA, which were synthesized by using a custom-made 3D printer. The printing method is based on the photo-polymerization technique, where a common projector coupled with a PC was used for this aim. The structural and morphological features of the 3D-printed filter were studied using X-ray diffraction and scanning electron microscopy, respectively. Once printed, the interaction of Hg(II) ions and the filters was studied by X-ray Photoelectron Spectroscopy (XPS) and Fourier Transform Infrared (FT-IR). Finally, the kinetics of interaction and the adsorption capacity at different temperatures were studied. The system, consisting of silver nanoparticles and a photopolymerizable monomer (PEGDA), represents one of the few systems that involve the use of noble metal nanostructures for the removal of heavy metals from water [66,67,68,69,70] and, in addition, the only one that involves a three-dimensional structuring of the filter.

## 2. Materials and Methods

### 2.1. Chemicals

Silver nitrate (AgNO_3_), sodium citrate (Na_3_C_6_H_5_O_7_, cit, 99% pure), L-cysteine (C_3_H_7_NO_2_S, Lcys, 97% pure) sodium borohydride (NaBH_4_, ≥98.0%), Poly(ethylene glycol) diacrylate (PEGDA, Mn = 700 g/mol, density 1.12 g/mL at 25 °C) and lithium phenyl-2,4,6-trimethylbenzoylphosphinate (LAP, ≤95% pure) were purchased from Merck (Darmstadt, Germany). They were used without further purification processes and dissolved in deionized water (18.2 MΩ·cm).

### 2.2. Synthesis of Silver Nanoparticles (AgNPs-cit-Lcys)

Silver nanoparticles capped with citrate and L-cysteine (AgNPs-cit-Lcys) were synthesized according to previous work [71]. Briefly, 10 mL of citrate solution [0.01 M in deionized water (dH_2_O)], 25 mL of L-cysteine solution (0.002 M in dH_2_O), and 2.5 mL of silver nitrate (AgNO_3_) solution (0.05 M in dH_2_O) were sequentially added in a 100 mL flask under stirring. AgNO_3_ is the precursor salt of Ag^+^ ions, while citrate and L-cysteine are the capping agents, which avoid the aggregation of growing silver nanoparticles. The mixture was then degassed with Argon for 10 min (this was accomplished at low pressure and room temperature with a cannula in the flask closed with a rubber stopper, allowing air to flow outside through another needle) and 4 mL of a sodium borohydride (NaBH_4_) solution (0.016 g in 4 mL of dH_2_O) added for reducing the Ag^+^ ions into Ag^0^ (nanoparticles). The mixture was allowed to react at room temperature (r.t.) for 2 h, and then, the brown solution was collected. The solution was stored in the fridge at T = 4 °C before use in the synthesis of the 3D filters.

### 2.3. Synthesis of PEGDA/AgNPs-cit-Lcys Filters by Using 3D Printer

**Figure 1:**
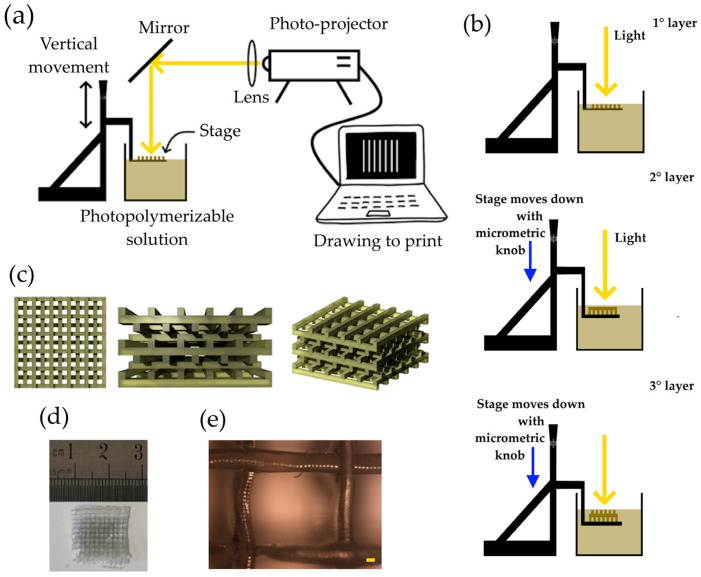
(**a**) schematic representation of 3D printer; (**b**) working principle of the 3D printer based on photopolymerization; (**c**) schematic drawings of a filter observed from different angles; (**d**) picture of a filter; and (**e**) image of optical microscope of a PEGDA/AgNPs-cit-Lcys filter (magnification 5×, scale bar 100 µm).

A photopolymerizable solution was synthesized by mixing PEGDA (30% in volume), AgNPs-cit-Lcys (70% in volume at the concentration of 0.5 mg/mL), and LAP (1% in weight with respect to the PEGDA mass). PEGDA was diluted in AgNPs-cit-Lcys, and LAP was added. The mixture was stirred for 20 min in the dark, and the solution was ready to be polymerized (the viscosity of the solution was 40.2 mPa∙s). Figure 1a shows the schematic representation of the 3D printer. A PC connected to a commercial photo-projector (Acer, Taipei, Taiwan, X1385WH, power and density of 3.30 mW and 106 mW/cm^2^, respectively, at 500 nm measured on the solution surface), a collimator lens, and a dichroic mirror (only the range 450–600 nm is reflected) composes the main part of the 3D printer. A metallic plate fixed on an XYZ translation stage and a plastic container complete the printer setup. The photopolymerization solution is poured into the plastic container to be polymerized on the surface of the metallic plate. The light coming from the photo projector passes through a lens (f = 201.0 mm), and the beam is reflected at 90° by the mirror. The metallic plate is immersed in the solution of PEGDA and AgNPs, and the knob can move it vertically. The light beam, once reflected, arrives onto a thin layer of the photopolymerizable solution, and the polymerization occurs only where the light is present. After the first layer is synthesized, the plate is moved down into the solution, through the micrometric knob, and a new layer is available to be polymerized (second layer). Among the exposition of the layers, the light beam is stopped (projecting a black slide), avoiding unwanted polymerizations. A schematic drawing of the working principle of 3D printing is shown in Figure 1b.

Figure 1c shows the schematic aspect of the final filter at different views. Parallel lines in one direction define the first layer. The second one is perpendicular with respect to the first, and the third layer is parallel to the first one but slightly shifted in one direction to create a mismatching of these two layers. The same idea is applied to the fourth layer, etc. By repeating the procedure, it is possible to make the desired structure. This kind of structure increases the surface area of the filter and favors the interaction between the metal ions and the adsorbent material.

Figure 1d shows a picture of a final hydrogel, while Figure 1e depicts an optical microscope image (Nikon L-IM coupled with a Nikon camera, model DS-U1), where lines have a length (thickness) of about 200 µm. Other optical images are reported in Figure S1 of the Supporting Information.

The exposure time for each layer was 1 min, the number of layers in a filter was 10, and the distance between two adjacent layers was 300 µm.

Before the investigations, the hydrogels were cleaned in a solution of water for 72 h at room temperature and in the dark, with a changing of the water every 24 h to remove any unreacted LAP and/or PEGDA molecules. The hydrogels were used for the filtering tests without any further process.

Although the mechanical properties of the material are not strictly necessary for the purpose of the following work, preliminary investigations were carried out through stress–strain measurements obtaining a Young’s modulus equal to 14 MPa ± 2 MPa. Further investigations need to confirm this result.

### 2.4. Kinetics of Interaction, Filtering Tests, and Adsorption Thermodynamics

The interaction kinetics were carried out in a static condition and at room temperature (T = 25 °C). Two 3D filters (total mass on average 0.4 g) were immersed in a beaker containing 10 mL of Hg(II) polluted water at 8 mg/L for different times (from 0 to 24 h). The mercury concentration was measured using inductively coupled plasma-optical emission spectroscopy (ICP-OES). The minimum time to reach the maximum adsorption was estimated to be ~8 h of interaction. Subsequently, the Hg(II) filtering tests were carried out by changing the Hg(II) ions concentration from 2 mg/L to 20 mg/L for three different temperatures: T_1_ = 4 °C, T_2_ = 25 °C, and T_3_ = 50 °C. To evaluate and compare the adsorption capacities, the amount of metal ion adsorbed per unit mass of the sorbent (mg/g) was measured by using Equation (1) [72]:

(1) $$q_{e}=\left(\frac{C_{i}-C_{e}}{m}\right)\times V$$

where *q_e_* (mg/g) is the adsorption capacity at equilibrium, *C_i_* and *C_e_*, are the initial and equilibrium concentrations of metal ion (mg/L), respectively, *V* is the volume of adsorbate solution in liters, and *m* is the mass of the filter in gram. The *C_i_* and *C_e_* were measured by ICP-OES. The removal efficiency (*RE*) was calculated according to the Equation (2) [72]:

(2) $$RE\;\left(\%\right)=\frac{\left(C_{i}-C_{e}\right)}{C_{e}}\times 100$$

The release of Ag from the filter was also studied. The washing waters of filters were collected every 24 h for 3 days and, subsequently, the Ag concentration was measured by ICP-OES. The Ag-releasing tests showed that no silver was lost during the investigation, revealing that the AgNPs were firmly incorporated into the polymer matrix.

The adsorption thermodynamics were evaluated following the procedure shown in the literature [73].

### 2.5. Analytical Greenness Metric Calculation

According to the idea of Pena-Pereira F. and co-workers [74], we calculated the Analytical GREEnness metric (AGREE); this parameter focuses on making analytical procedures more environmentally benign and safer for humans. A detailed description of the AGREE metric is reported in the Supporting Information. Based on the 12 parameters, a score of 0.42 was calculated, which is in line with most of the literature [75,76,77,78,79].

### 2.6. Instrumentation

An Ultraviolet/Visible/Near infrared (UV/Vis/NIR) spectrophotometer (Lambda 750, Perkin Elmer, Shelton, CT, USA) was used to optically characterize the samples in the range of 300–700 nm.

The concentrations of Hg and Ag in the water were measured by ICP-OES (Avio 200, Perkin-Elmer). After a suitable calibration, Hg and Ag concentrations were analyzed using the 253.652 nm and 328.068 nm emission lines, respectively. All the tests were repeated three times. Detailed information about the calibration of the ICP-OES instrument is reported in the Supporting Information.

The viscosity of the photopolymerizable solution was measured by AR 2000ex rheometer (TA Instruments, New Castle, DE, USA), using a steady-state flow, applying a shear rate of 1.000 s^−1^, and the measures were in triplicate.

A Philips X-Pert Pro 500 (Amsterdam, The Netherlands) diffractometer X-ray diffraction (XRD) was performed using Cu Kα radiation (λ = 1.54056 Å) in the 5–90° 2θ range, with a 1 s counting time and 0.02° step size. A 3D-printed filter was first dried in an oven at 120 °C for 24h to remove completely the solvent, and then, the sample was ground to a fine powder. The morphology of the samples was investigated using a Zeiss Leo SUPRA™ 35 (Oberkochen, Germany) field emission scanning electron microscope (FE-SEM). SEM images are shown in the Supporting Information in Figure S2, while Figure S3 shows the XRD spectrum of a filter.

Fourier transform infrared (FT-IR) measurements were performed by a VECTOR 22 (Bruker, Billerica, MA, USA) FT-IR interferometer equipped with a deuterated triglycine sulfate detector operating in between 400 and 4000 cm^−1^, with a resolution of 1 cm^−1^. Reflection–absorption infrared spectroscopy (RAIRS) measurements were carried out with a Specac P/N 19,650 series monolayer/grazing angle accessory; spectra were collected at incidence angles of 70° with respect to the normal to the sample surface. 

X-ray photoelectron spectroscopy (XPS) probed the chemical composition and molecular structure of the filters. XPS measurements were carried out on the PEGDA membranes doped with AgNPs before and after Hg(II) filtering tests. XPS analysis was performed with a custom-made instrument, having two separate chambers: one for the samples preparation and the second one for analyzing them in UHV (ultra-high vacuum). The latter is equipped with a six-degree-of-freedom manipulator and a hemispherical electron analyzer (mean radius = 150 mm) with a five-lens output system combined with a 16-channel detector, which gives a total instrument resolution of 1.0 eV as measured at the Ag 3d_5/2_ core level. Samples were degassed overnight into the preparation chamber, until the total pressure was about 10^−8^ Torr, before introducing them in the analysis chamber. A 10^−8^–10^−9^ Torr range was the typical vacuum pressure in the analysis chamber during the measurements. The non-monochromatized Mg K*α* (1253.6 eV) was used as the X-ray radiation source. Calibration of the energy scale was accomplished by referencing the spectra to the C1s core level signal of aromatic C atoms, found at 284.70 eV, for all samples. Atomic ratio values were calculated from peak intensities divided by Scofield’s factors [80]. After subtraction of a Shirley-type background, the C1s, N1s, O1s, S2p, and Ag3d spectra were fitted by using Gaussian profiles as fitting functions. The S2p_3/2, 1/2_ doublets were fitted using the same full width at half-maximum (FWHM) for both components, a spin−orbit splitting of 1.2 eV, and a branching ratio (2p_3/2_/2p_1/2_) of 2. The Ag3d_5/2, 3/2_ doublets were fitted using the same FWHM for both components, a spin−orbit splitting of 6.0 eV, and a branching ratio (3d_5/2_/3d_3/2_) of 3/2. For the Hg4f_7/2, 5/2_ doublets, a spin−orbit splitting of 4.1 eV and a branching ratio (4f_7/2_/4f_5/2_) of 4/3 was selected. When several different species were identified in a spectrum, the same FWHM value was set for all single photoemission bands.

## 3. Results and Discussion

### 3.1. Optical Characterization

**Figure 2:**
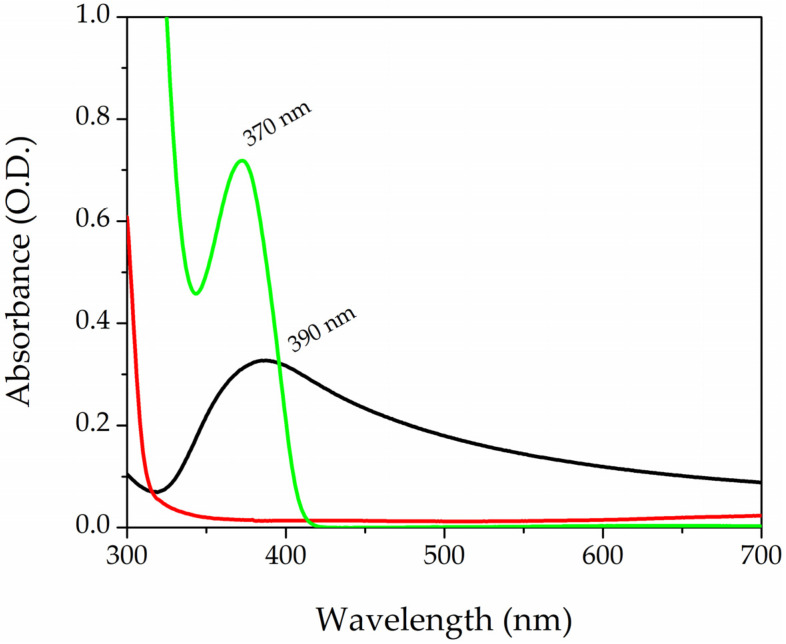
UV-Vis absorption spectra of AgNPs-cit-Lcys (black curve) colloidal solution, PEGDA700 (red line), and LAP solution at the concentration of 0.1% in wt (green curve).

Figure 2 shows the absorption spectra of the three components of the photopolymerizable solution. The black curve refers to the AgNPs-cis-Lcys colloidal solutions, while the red and green lines correspond to PEGDA and LAP, respectively. AgNPs have a surface plasmon resonance (SPR) band centered at 390 nm, with an FWHM of 120 nm; these particles have a spherical shape and a mean diameter of 5 nm, as reported in the literature [71]. The PEGDA spectrum does not show any peaks in that range of wavelength. On the contrary, the LAP solution has a band peaked at 370 nm. The tail of the photoinitiator enters into the visible range, and this organic molecule is divided into radicals by exposition to visible light and starts the photopolymarizable reaction by opening the double bonds of the acrylate ends (R-C=C) of the PEGDA. Two PEGDA chains join together, forming a covalent bond R-C-C-R’ (yield of polymerization reaction was 90%, the detailed calculation is in the Supporting Information). A sketch of the process is reported in Figure S4 of the Supporting Information.

### 3.2. Molecular Structure and Chemical Composition: FT-IR and XPS Studies

**Figure 3:**
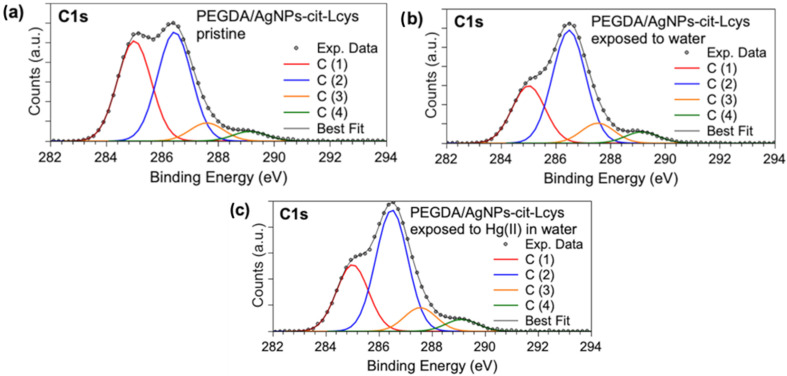
XPS C1s core-level spectra collected on (**a**) pristine PEGDA/AgNPs-cit-Lcys, (**b**) PEGDA/AgNPs-cit-Lcys after immersion in water, (**c**) PEGDA/AgNPs-cit-Lcys after immersion in water containing 20 mg/L Hg(II).

X-ray photoelectron spectroscopy measurements allowed for investigating the molecular structure and chemical composition of PEGDA filters enriched with silver nanoparticles. The core-level spectra acquired at the C1s, S2p, Ag3d, and Hg4f allowed for ascertaining the presence and stability of AgNPs-cit-Lcys on the surface of the PEGDA membranes, and they gave information about the presence of Hg after the filtering activity, as well as useful information about the heavy metal oxidation state. The binding energy (BE), FWHM, atomic ratio values, and proposed assignments for all the measured signals are reported in Table S1 in the Supporting Information; here, only the most indicative signals will be discussed. C1s XPS spectra (Figure 3a–c) display four components assigned, respectively, to aliphatic and aromatic C (C1, BE = 285.0 eV) and C-S of L-cysteine, C-N and C-O (C2, BE = 286.5 eV), C=O (C3, BE = 287.6 eV), and COOR (R = H, C) (C4, BE = 289.1 eV) functional groups. The relative intensities of C1s spectral components are stable in the three samples (see Table S1), confirming the chemical stability of the PEGDA/AgNPs-cit-Lcys membranes; the lowering of the C1 component intensity after the membranes’ immersion in water is probably due to the removal of the C-C contaminants that are always observed in samples prepared in air [81,82].

**Figure 4:**
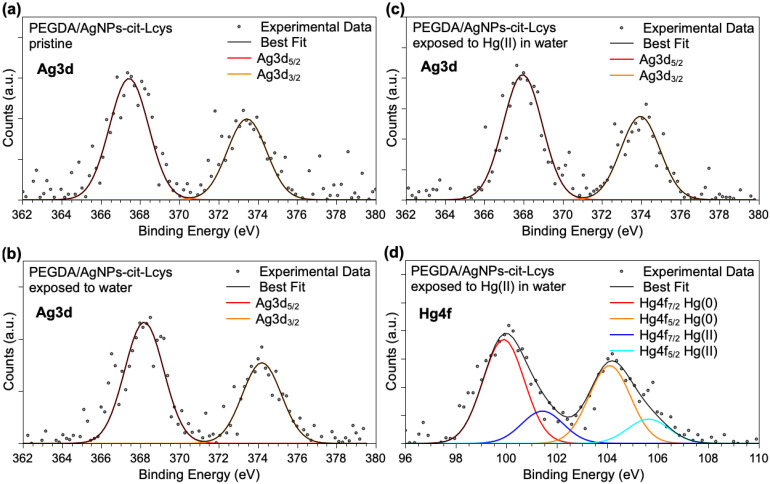
XPS Ag3d core-level spectra collected on (**a**) pristine PEGDA/AgNPs-cit-Lcys, (**b**) PEGDA/AgNPs-cit-Lcys after immersion in water, (**c**) PEGDA/AgNPs-cit-Lcys after immersion in water containing 20 mg/L Hg(II), (**d**) Hg4f spectrum collected on PEGDA/AgNPs-cit-Lcys after immersion in water containing 20 mg/L Hg(II).

Ag3d and Hg4f spectra are shown in Figure 4. It is noteworthy that Ag3d spectra are analogous for the three samples, confirming the NPs’ stability in the PEGDA membrane also after immersion in water and exposure to Hg(II) ions. However, due to the very low intensity of the signals, the components individuated by applying a peak-fitting procedure would not be reliable, and only a main signal indicative for Ag atoms can be unambiguously pointed out (Ag3d_5/2_ BE = 368 eV). The Hg4f spectrum reported in Figure 4d as measured on the PEGDA/AgNPs-cit-Lcys after immersion in water containing 20 mg/L Hg(II) suggests that mercury ions are partially reduced by the interaction with silver nanoparticles, as already observed for similar systems. Actually, two spin–orbit pairs are observed: the first signal (Hg4f_7/2_ BE = 99.9 eV) is due to atomic Hg(0), probably forming an alloy with silver atoms, and the peaks at higher BE (Hg4f_7/2_ BE = 101.4 eV) are indicative for the presence of Hg(II) ions. This finding is not surprising, since in a previous work, we investigated in detail the interaction between free-standing hydrophilic silver nanoparticles and Hg(II) ions in water samples, revealing a redox activity leading to reduced Hg(0) and aggregated particles of mixed Hg(0)/Ag(0) [83]. This also suggests that PEGDA does not modify the AgNPs-Hg interaction mechanism.

**Figure 5:**
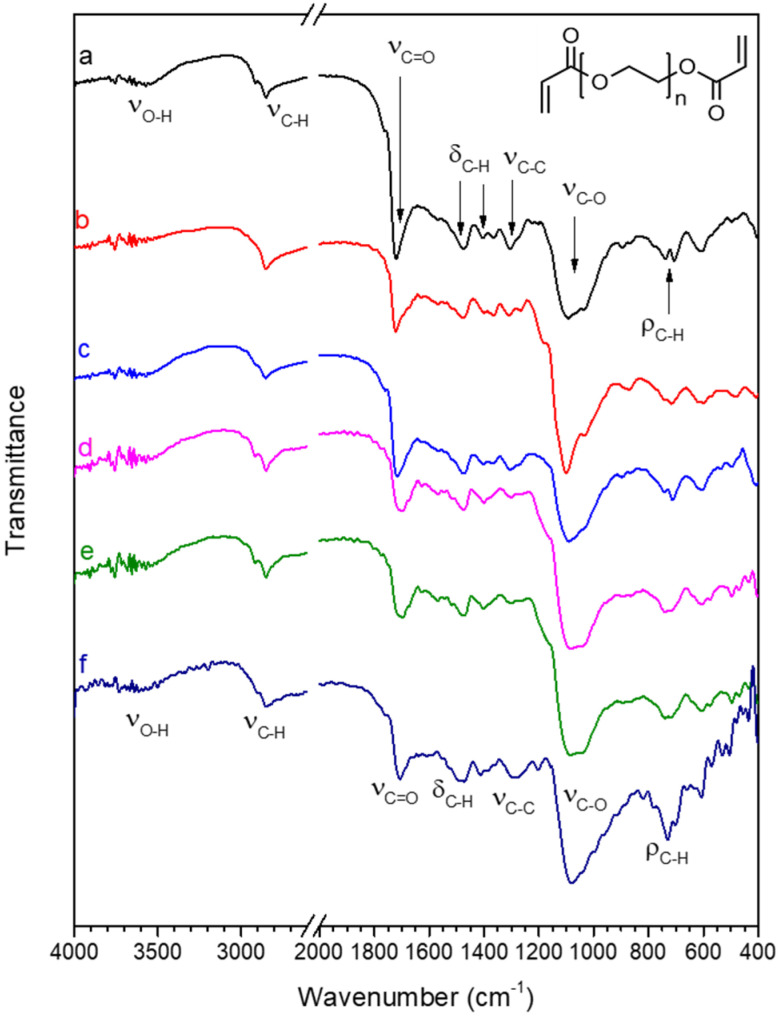
FT-IR spectra in the 4000–2600 and 2000–400 cm^−1^ range of (a) pristine PEGDA; (b) PEGDA after 24 h immersion in water; (c) PEGDA after 24 h immersion in water containing 20 mg/L Hg(II); (d) PEGDA/AgNPs-cit-Lcys; (e) PEGDA/AgNPs-cit-Lcys after 24 h immersion in water; (f) PEGDA/AgNPs-cit-Lcys after immersion in water containing 20 mg/L Hg(II). The chemical structure of PEGDA is also shown in the top right corner of the figure.

The FT-IR spectrum of pristine PEGDA, shown in Figure 5a, is in agreement with the spectra reported in the literature [84,85]; for clarity, Table 1 reports a summary of the main peak’s position and assignment. In the spectrum of PEGDA, most peaks are related to the PEG backbone. The peaks at 2920 and 2850 cm^−1^ (ν_C-H_) are related to the stretching vibrations of aliphatic C-H bonds, peaks at 1470 and 1370 cm^−1^ (δ_C-H_) are due to bending vibrations of aliphatic C-H, and the peaks at 710–730 cm^−1^ (ρ_C-H_) to rocking vibrations of the methylene group. The most intense peak in the PEG spectrum is related to the C-O stretching vibration (ν_C-O_ 1090 cm^−1^), located in Figure 5a. The chemical structure of the PEGDA polymer shows two ester functional groups at the chain terminal, producing an intense C=O stretching peak (ν_C=O_), located at 1725 cm^−1^ in Figure 5a, and a lower intensity peak at about 1300 cm^−1^ (ν_C-O_), related to stretching vibrations of C-O bonds that are part of the ester function [86]. Since the C=O stretching vibration (ν_C=O_) is due to the chain terminals and the PEG-type C-O stretching vibration (ν_C-O,_ 1090 cm^−1^) to the chain backbone, the higher the intensity ratio between ν_C=O_ and ν_C-O_, the shorter the chain length of the PEG backbone. A low-intensity O-H stretching peak at about 3600 cm^−1^ (ν_O-H_) can be due to physisorbed water.

Treatment with water does not alter the structure of PEGDA, since in the spectrum of PEGDA after 24 h immersion in water (spectrum b), peaks are found in the same position of the pristine sample spectrum. The spectrum of PEGDA after immersion in water containing Hg(II) (spectrum c) also shows that the structure of PEGDA remains unaltered by the treatment.

The incorporation of silver nanoparticles in the PEGDA membrane to produce sample PEGDA/AgNPs-cit-Lcys (spectrum d) does not alter the chemical structure of PEGDA, and the PEGDA/AgNPs-cit-Lcys membrane appears stable even after immersion in pure water (spectrum e) and in water containing Hg(II) (spectrum f). These results confirm that the Hg(II) ions do not preferentially interact with PEGDA chains.

### 3.3. Effect on Mercury Ions Concentration: Adsorption Isotherms

**Figure 6:**
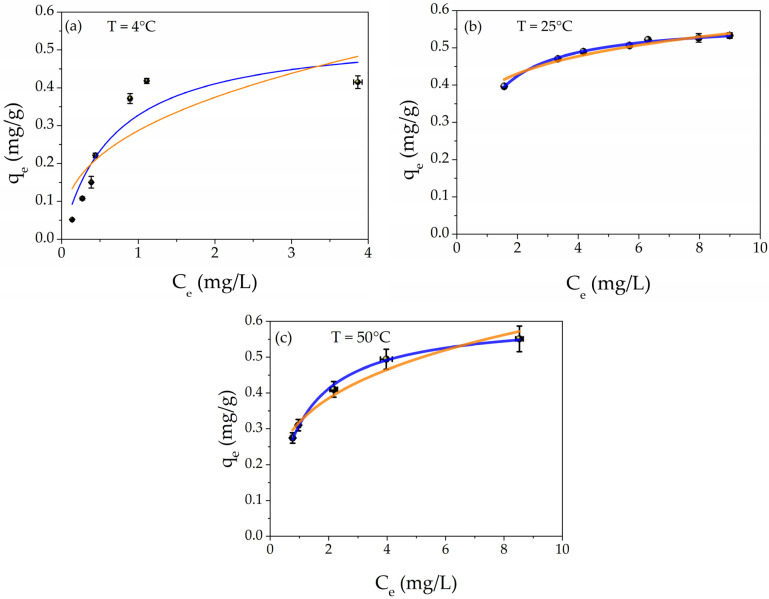
Plot of adsorption capacity (*q_e_*) as a function of equilibrium concentration *C_e_* at different temperatures: (**a**) T = 4 °C, (**b**) T = 25 °C, and (**c**) T = 50 °C; the isotherm best fits are also shown in the graphs: orange line Freundlich and blue line Langmuir models, respectively.

The adsorption capacity study was carried out by using different aqueous concentrations of Hg(II) from 2 to 20 mg/L. Figure 6 depicts the adsorption capacities (black points) as a function of the Hg(II) equilibrium concentration at different temperatures T = 4 °C, T = 25 °C, and 50 °C, respectively. In addition, the best fit of the experimental points is presented. The orange line is the Freundlich isotherm; meanwhile, the blue curve represents the Langmuir model.

The Freundlich isotherm is defined by Equation (3) [87]:

(3) $$q_{e}=K_{F}\times C_{e}^{\frac{1}{n}}$$

where, *K_F_* is Freundlich’s coefficient and is related to the adsorption capacity, while *n* is a number related to the strength constant of the isotherm model. 

The Langmuir isotherm is defined according to Equation (4) [87]:

(4) $$q_{e}=\frac{q_{m}\times K_{L}\times C_{e}}{1+K_{L}\times C_{e}}$$

where *q_m_* (mg/g) is the maximum adsorption capacity and *K_L_* (L/mg) is the Langmuir isotherm constant. The *q_e_* and *C_e_* parameters have the same meaning as those in Equation (1). The chemo-physical meanings of the two isotherms are explained in the literature [87,88]. Briefly, the Freundlich isotherm model describes the physisorption process, while the Langmuir isotherm refers to the chemisorption phenomenon. 

The parameters obtained by the best fits are listed in Table 2 for all temperatures. For each temperature, it is possible to notice that the R^2^ values of Langmuir isotherms are higher than those of Freundlich models, confirming the chemisorption nature of the mechanism. In addition, the maximum adsorption value (*q_m_*) increases with the increasing temperature, from 0.55 mg/g to 0.61 mg/g for 4 °C and 50 °C, respectively. This result indicates that the enhancement of the provided energy encourages the adsorption of Hg(II) into the metal core of AgNPs, forming the amalgam Hg/Ag, as reported in the XPS study and our previous research [68,83]. A comparison with the literature [66,67,68,69,70] about the performance of filtration toward heavy metal ions is reported in Table S2 of the Supporting Information.

The removal efficiency was calculated according to Formula (2). For the temperatures of 4 °C, 25 °C, and 50 °C, they were 94%, 94%, and 86%, respectively; these values were determined by averaging the removal efficiencies over the range of 2–20 mg/L at the three different temperatures. The values show that the RE is almost constant in this temperature range (the calculated error was 5% as the standard deviation).

### 3.4. Adsorption Thermodynamics

**Figure 7:**
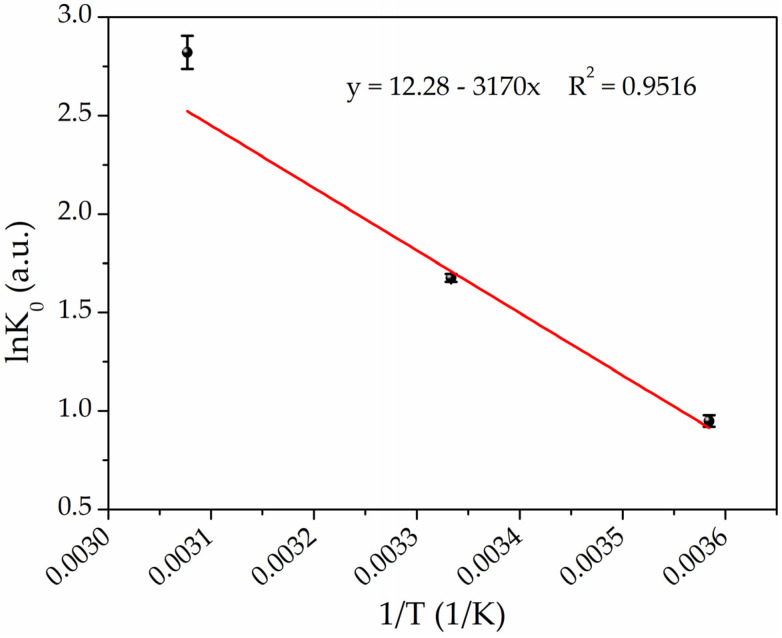
Van’t Hoff plot for the adsorption of Hg(II) on PEGDA/AgNPs-cit-Lcys at the temperatures of 4 °C, 25 °C, and 50 °C (black points refer to experimental data, while red line represents the best fit).

The thermodynamic parameters were calculated by following the method described in the literature [73,89]. Briefly, the adsorption isotherms at the three temperatures were plotted as *ln*(*q_e_*/*C_e_*) as a function of *q_e_*. Subsequently, linear regressions were performed to determine the intercept, which represents the *lnK*_0_, where *K*_0_ is the thermodynamic equilibrium constant. Δ*G*_0_ was calculated by following Equation (5) [73]:

(5) $$∆G_{0}=-RTlnK_{0}$$

where *R* is the universal gas constant [8.314 J/(mol·K)], and T is the temperature in Kelvin. Subsequently, by plotting *lnK*_0_ versus 1/T (Van’t Hoff plot) as shown in Figure 7, it is possible to determine the enthalpy (Δ*H*_0_) and entropy (Δ*S*_0_) changes from the slope and intercept, respectively, by fitting the experimental data according to Equation (6) [73]:

(6) $$lnK_{0}=\frac{∆S_{0}}{R}-\frac{{∆H}_{0}}{RT}$$

The thermodynamic parameters are listed in Table 3. The negative values of Δ*G*_0_ confirm that the adsorption is spontaneous, and the negative increases of Δ*G*_0_ values with the increasing temperature indicate that the adsorption reactions are thermodynamically more favorable at higher temperatures. The observed positive enthalpy change (Δ*H*_0_ = 26.36 kJ/mol) confirms that the adsorption process is endothermic, and the positive Δ*S*_0_ value [102.09 J/(mol·K)] indicates that the adsorption is driven by entropy change, which means that the nature of the adsorbate or adsorbent has been changed in the process of adsorption (amalgamation) [89,90].

### 3.5. Effect on Contact Time: Adsorption Kinetics 

**Figure 8:**
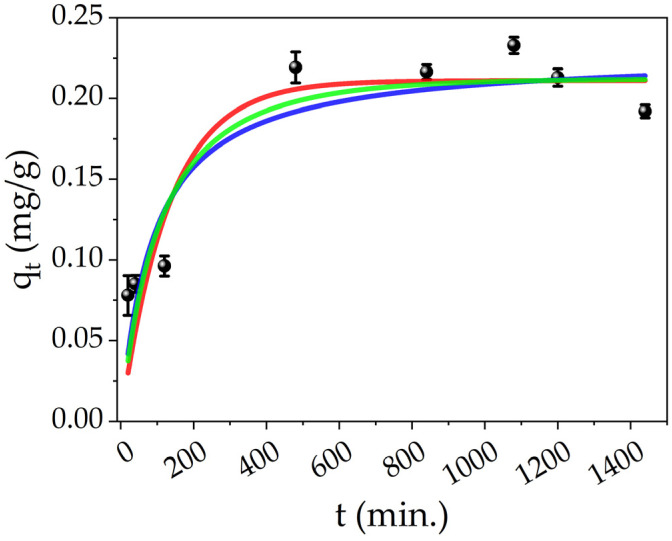
Effect of contact time on the Hg(II) adsorption onto PEGDA/AgNPs-cit-Lcys (black points), fittings of different kinetic models for Hg(II) adsorption on PEGDA/AgNPs-cit-Lcys: PFO (red line), PSO (blue line) and MO (green line).

The effect on contact time was studied by measuring the concentration of Hg(II) ions of the treated solutions at different times. Figure 8 shows the adsorption data of Hg(II) by PEGDA doped with AgNPs-cit-Lcys at different times (black points), and the adsorption capacity reaches the equilibrium value (q_e, exp._ = 0.21 mg/g, average value of the last five points) in about 8 h. The experimental data were fitted by using different kinetic models: pseudo-first order (PFO, red line), pseudo-second order (PSO, blue line), and mixed order (MO, green line) developed by X. Guo and J. Wang [91,92]. The analytical formulas of these models are collected and explained in detail in the literature [91,92]. To understand the adsorption kinetics of our system, the conditions of applicability of the models have to be considered. For the PFO, three conditions can occur: the initial concentration of pollutant is higher than the active sites, at the initial stage of adsorption, the occupation of active sites of the adsorbent is almost zero, and finally, the number of active sites is low. Also in the case of the PSO, three conditions can describe the process: the initial concentration of pollutant is low, the final step of the process is the adsorption on the active sites, and the number of active sites is higher than the ions [92]. These two models only describe some particular conditions and not the overall adsorption process which can be more complex; indeed, the conditions of a real adsorption process change during the process itself. On the contrary, the MO model takes into account the entire adsorption process and it can represent the conditions at any stage of the adsorption process. Moreover, the limiting step can be the diffusion of the ions and/or the adsorption of them on the active sites, and finally, the pollutant’s initial concentration is arbitrary.

**Figure 9:**
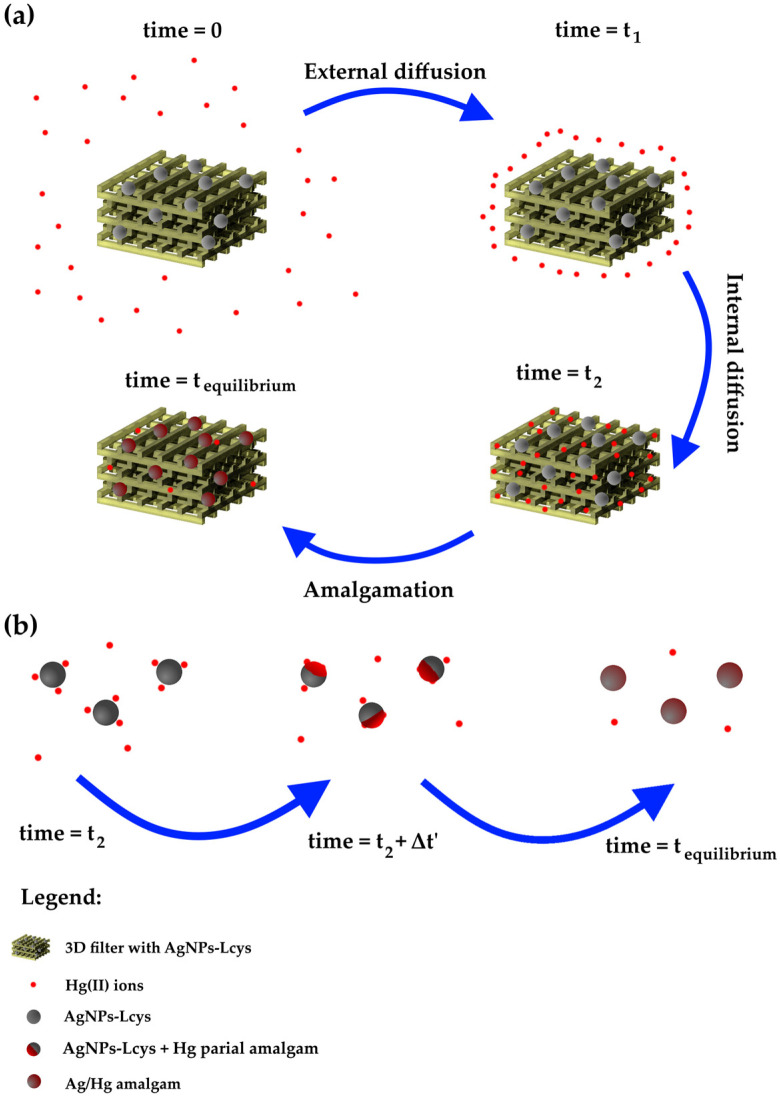
Schematic representation of (**a**) the overall adsorption process; (**b**) the actual chemisorption process occurring between AgNPs-Lcys and Hg(II) ions.

The fitting parameters and the experimental adsorption capacity are listed in Table 4. All the models show an excellent approximation of the *q_e_*, while the R^2^ value slightly increases from the PFO to MO model and suggests that the MO is the appropriate kinetic model. In order to understand the chemical meaning of the interaction, the mathematical interpretation of the chemical one is also added. As reported in our previous works [71,83], the colloidal solutions of AgNPs interact by reducing the Hg(II) ions in Hg(0) and forming a mercury–silver amalgam within several minutes. Meanwhile, from Figure 8, it is clear that it is necessary at least 8 h to complete the adsorption (and thus the interaction); consequently, the limiting step can be the internal diffusion of the ions in the PEGDA. Referring to Figure 9a, at the time zero, the active sites are completely empty. In the figure, the grey spheres represent the silver nanoparticles (active sites), the red points refer to mercury ions, and the yellow 3D structure is the PEGDA filter. In the subsequent stage, the Hg(II) ions reach the surface of the filter (external diffusion, time = t_1_ in the picture), and then, the ions arrive at the AgNP surface by internal diffusion (time = t_2_). Once reached by the silver particles, the amalgamation occurs, represented by the dark red spheres in the picture. Finally, the process finishes when all the silver nanoparticles adsorb their maximum amount of mercury (time = t_equilibrium_). More specifically, the chemisorption process is shown in Figure 9b. The mercury ions diffuse through the polymer matrix and arrive near the AgNPs. The mercury ions interacting with the surface of the AgNPs are reduced. The formation of the Ag/Hg amalgam starts (t_2_ + Δt’), until complete amalgamation (t_equilibrium_) is accomplished, and the maximum chemisorption of mercury by the silver nanoparticles is fulfilled.

## 4. Conclusions

In this work, the insertion of AgNPs-cit-Lcys into a PEGDA matrix by a 3D printing process has been proven. The XPS and FT-IR measurements showed that the PEGDA did not interfere with the AgNPs’ chemical interaction with the Hg(II) ions in the subsequent Hg/Ag amalgam formation. The adsorption-capacity studies revealed that the adsorption process is chemisorption (Langmuir model), and by increasing the temperature of interaction (from 4 °C to 50 °C), the maximum adsorption increases (from 0.55 to 0.61 mg/g). The removal efficiency was evaluated for the three temperatures, obtaining for 4 °C, 25 °C, and 50 °C the values 94%, 94%, and 86%, respectively. Despite the good results, this type of system presents some limitations, such as relatively long filtering times (8 h) and limited maximum adsorption capacity (0.57 mg/g at 25 °C). In addition, we limited the study of the 3D filters only to static conditions. Nevertheless, we are confident that the systems can work with additional improvements on the doping of the matrix and the mechanical properties in dynamic conditions (water flow and/or under pressure), giving better removal efficiency and reducing the filtering times.

## Figures and Tables

**Table 1:** Position (in cm^−1^) and assignment of the main peaks in the FT-IR spectra are shown in Figure 5.

Peak	ν_O-H_	ν_C-H_	ν_C=O_	δ_C-H_	ν_C-C_	ν_C-O_	ρ_C-H_
Position (cm^−1^)	3600	2960 2850	1725	1470 1370	1300	1090	710 730
Peak assignment	O-H stretching	C-H stretching	C=O stretching	C-C bending	C-C stretching	C-O stretching	C-H rocking

**Table 2:** Isotherm parameters determined by fitting the experimental points.

Interaction Temperature	Isotherm Model	Parameters
T = 4 °C	Freundlich	*K_F_* = 0.29 ± 0.04 mg/g
		*n* = 2.6 ± 0.8
		R^2^ = 0.644
	Langmuir	*K_L_* = 1.5 ± 0.6 L/mg
		*q_m_* = 0.55 ± 0.09 mg/g
		R^2^ = 0.841
T = 25 °C	Freundlich	*K_F_* = 0.30 ± 0.01 mg/g
		*n* = 3.1 ± 0.7
		R^2^ = 0.942
	Langmuir	*K_L_* = 1.42 ± 0.05 L/mg
		*q_m_* = 0.57 ± 0.01 mg/g
		R^2^ = 0.994
T = 50 °C	Freundlich	*K_F_* = 0.32 ± 0.01 mg/g
		*n* = 3.7 ± 0.4
		R^2^ = 0.949
	Langmuir	*K_L_* = 1.04 ± 0.06 L/mg
		*q_m_* = 0.61 ± 0.01 mg/g
		R^2^ = 0.995

**Table 3:** Thermodynamic parameters of the Hg(II) adsorption onto PEGDA/AgNPs-cit-Lcys at different temperatures.

Temperature (°C)	*lnK*_0_	Δ*G*_0_ (kJ/mol)	Δ*S*_0_ [J/(mol·K)]	Δ*H*_0_ (kJ/mol)
4	0.95 ± 0.03	−2.20 ± 0.07	102 ± 3	26.4 ± 0.8
25	1.68 ± 0.02	−4.18 ± 0.13		
50	2.82 ± 0.08	−7.62 ± 0.23		

**Table 4:** Fitting parameters.

Kinetic Model		
Pseudo-first order	k_1_ (1/min)	0.008 ± 0.002
	q_e, calc._ (mg/g)	0.21 ± 0.01
	R^2^	0.858
Pseudo-second order	k_2_ (g/mg·min)	0.05 ± 0.02
	q_e, calc._ (mg/g)	0.23 ± 0.01
	R^2^	0.862
Mixed order	k_1_ (1/min)	0.004 ± 0.001
	k_2_ (g/mg·min)	0.03 ± 0.01
	q_e, calc._ (mg/g)	0.21 ± 0.02
	R^2^	0.871
	q_e, exp._ (mg/g)	0.21 ± 0.01

k_1_ kinetic constant of first order. k_2_ kinetic constant of second order. q_e, calc._ adsorption capacity at the equilibrium calculated by the fit.

## Data Availability

Data are contained within the article and Supplementary Materials.

## Supplementary Materials

The following supporting information can be downloaded at: https://www.mdpi.com/article/10.3390/polym16081034/s1, Figure S1. Optical microscopy of a filter section: (a) picture of 3 layers with layer 1 and layer 3 in the same focal plane; layer 2 is out of focus; (b) schematic representation of the 3 planes; (c) picture of 3 layers with a cross section of layer 2 in the focal plane and layer 1 and 3 out of focus; (d) schematization of the 3 layers; Figure S2. SEM images of 3D-printed filter at different magnification scale bars 20 µm and 1 µm for (a) and (b), respectively. A classic morphology of a polymer can be observed in the picture with a compact structure with very low porosity; Figure S3. XRD spectrum of a 3D-printed filter. The wide band at 20° confirms the amorphous nature of the PEGDA/AgNPs-cit-Lcys system; Figure S4. Photopolymerization reaction of PEGDA with the LAP photoinitiator; Table S1: Complete collection of XPS data; Figure S5. Result of the AGREE analysis for our analytical process; Table S2. Comparison of filtering capabilities toward heavy metal ions of various nanocomposite hydrogels with gold or silver metal nanostructures.
