# Supplementary material for: Three-Dimensional Printed Filters Based on Poly(ethylene glycol) Diacrylate Hydrogels Doped with Silver Nanoparticles for Removing Hg(II) Ions from Water
Source: Polymers (Basel). 2024 Apr 10;16(8):1034. doi: 10.3390/polym16081034 (PMC11054970; doi:10.3390/polym16081034)
Supplement: Supplementary file 1 [file polymers-16-01034-s001.zip › polymers-2868140-supplementary.pdf]

Supporting Information

Three-Dimensional Printed Filters Based on Poly(ethylene glycol) Diacrylate Hydrogels Doped with Silver Nanoparticles for Removing Hg(II) Ions from Water

Luca Burratti [1,*], Federica Bertelà [1], Michele Sisani [2], Irene Di Guida [2], Chiara Battocchio [1], Giovanna Iucci [1], Paolo Prosposito [3] and Iole Venditti [1]

[1] Department of Sciences, Roma Tre University of Rome, Via della Vasca Navale 79, 00146 Rome, Italy; federica.bertela@uniroma3.it (F.B.); chiara.battocchio@uniroma3.it (C.B.); giovanna.iucci@uniroma3.it (G.I.); iole.venditti@uniroma3.it (I.V.)
[2] Prolabin & Tefarm S.r.l., 06134 Perugia, Italy; michele.sisani@prolabintefarm.com (M.S.); irene.diguida@prolabintefarm.com (I.D.G.)
[3] Department of Industrial Engineering, University of Rome Tor Vergata, Via del Politecnico 1, 00133 Rome, Italy; paolo.prosposito@uniroma2.it
* Correspondence: luca.burratti@uniroma3.it

Optical microscopy

A filter (PEGDA/AgNPs-cit-Lcys) was dryed and cut along the cross section and it was observed under optical microscope. The Figure S1 shows the cross section of the filter. Figure S1a is a magnification of three layers, the blue lines highlight the layer position on the picture, the layer 1 and layer 3 are on the same focal plane, while the layer 2 is out of focus. Moreover, the layer 1 and 3 are parallel layers, meanwhile the layer 2 was printed perpendiculary with respect to the others, consequently, the cross section of this layer is coming out of the focal plane. Figure S1b rapresents a schematic drawing of the 3 layers. Figure S1c shows the same filter portion by changing the focus of the optical microscope, in this case, the section of layer 2 is on focus and the cut that has been made can be observed. Figure S1d schematizes the configuration of the three layers.

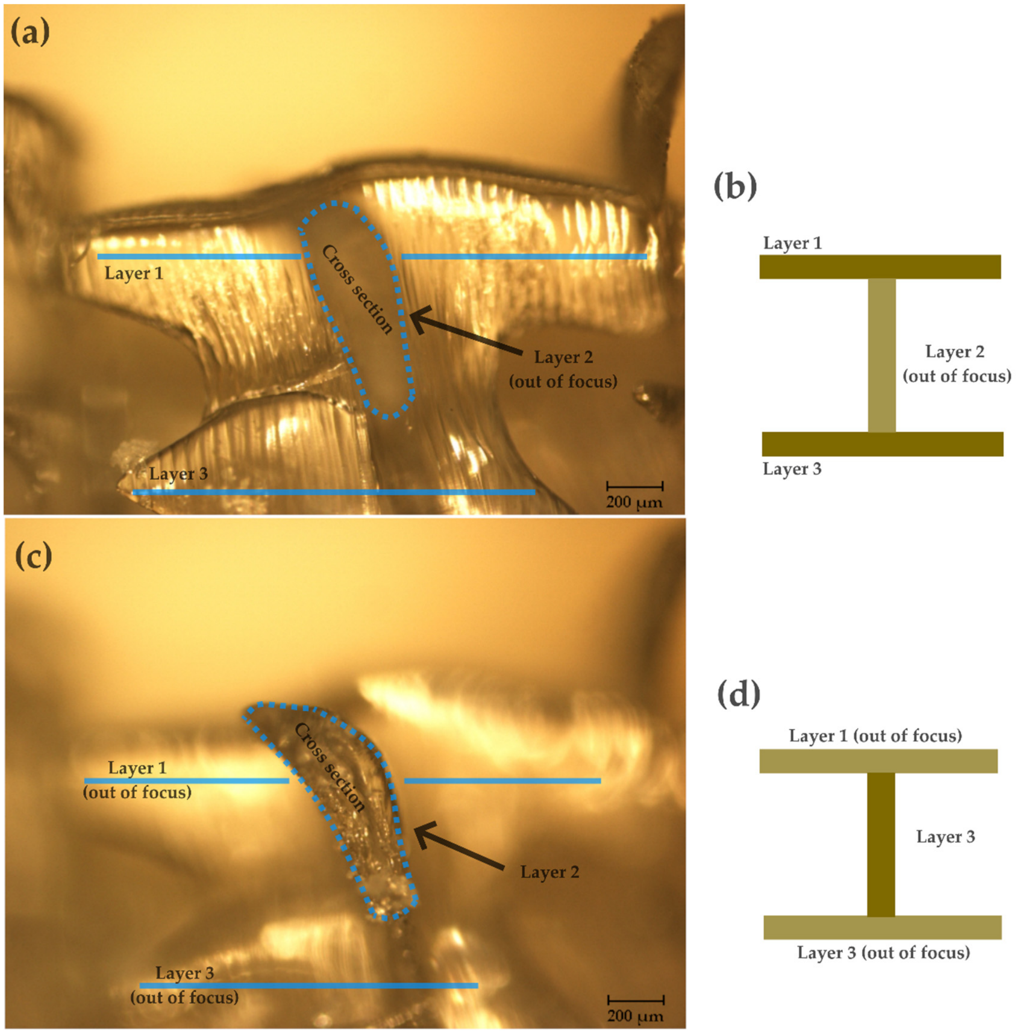

Figure S1. Optical microscopy of a filter section: (a) picture of 3 layers with the layer 1 and layer 3 in the same focal plane, layer 2 is out of focus; (b) schematic representation of the 3 planes; (c) picture of 3 layers with cross section of layer 2 in focal plane and layer 1 and 3 out of focus; (d) schematization of the 3 layers.

(a) (b)

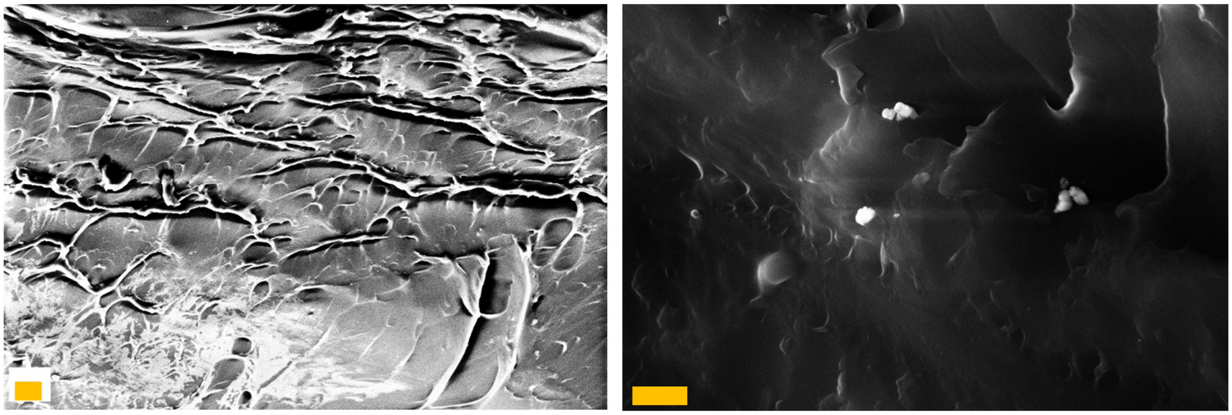

Figure S2. SEM images of 3D printed filter at different magnification scale bars 20 μm and 1 μm for (a) and (b), respectively. A classic morphology of a polymer can be observed in the picture with a compact structure with very low porosity.

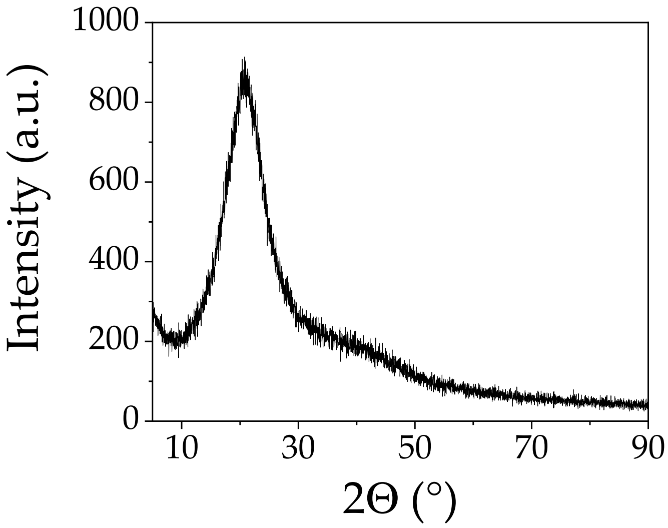

Figure S3. XRD spectrum of a 3D printed filter. The wide band at 20° confirms the amorphous nature of the PEGDA/AgNPs-cit-Lcys system.

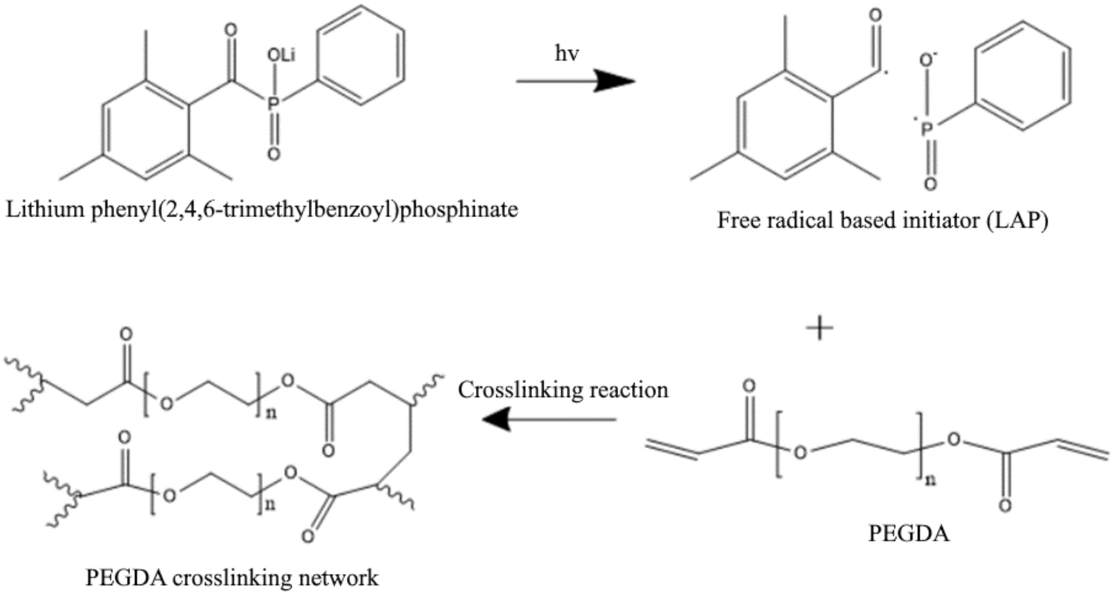

Figure S4. Photopolymerization reaction of PEGDA with the LAP photoinitiator.

Table S1. Complete collection of XPS data

Sample	Signal	BE (eV)	FWHM	Atomic %	Internal Atomic ratios	Assignments
PEGDA/AgNPs-cit-Lcys	C1s	285.00	1.42	65.56	10.3	C=C,C-C,C-S
		286.44			11.2	C-N,C-O
		287.59			1.9	C=O
		289.13			1.0	COOR
	O1s	532.77	1.75	28.00	7.6	C=O
		534.32			1.0	C-O, -OH
	N1s	398.51	2.13	0.11	1.0	$-NH_2$
	$S2p_{3/2}$	162.94	2.38	6.3	1.0	RS-Ag
		165.34			1.0	RS-H
	$Ag3d_{5/2}$	367.44	2.34	0.03	1.0	
PEGDA/AgNPs-cit-Lcys exposed to water	C1s	285.00	1.42	64.68	5.0	C=C,C-C,C-S
		286.48			10.0	C-N,C-O
		287.53			1.8	C=O
		289.15			1.0	COOR
	O1s	532.90	1.79	34.90	7.8	C=O
		534.48			1.0	C-O, -OH
	N1s	400.38	2.76	0.10	1.0	$-NH_2$
	$S2p_{3/2}$	164.59	2.15	0.30	0.8	RS-H
		170.87			1.0	RSOxidized
	$Ag3d_{5/2}$	368.19	2.34	0.02	1.0	
PEGDA/AgNPs-cit-Lcys exposed to Hg(II) in water	C1s	285.00	1.41	64.76	5.6	C=C,C-C,C-S
		286.49			10.2	C-N,C-O
		287.56			1.9	C=O
		289.12			1.0	COOR
	O1s	532.76	1.63	33.31	4.6	C=O
		534.07			1.0	C-O, -OH
		535.83				Phys. water
	N1s	397.97	2.43	0.86	1.1	$-NH_2$
		400.98			1.0	$-NH_3^+$
	$S2p_{3/2}$	160.14	2.05	0.97	1.1	RS-Ag
		162.78			1.6	RS-Ag
		166.66			1.0	RSOxidized
	$Ag3d_{5/2}$	367.95	2.34	0.03	1.0	
	$Hg4f_{7/2}$	99.89	1.93	0.07	3.2	Hg(0)
		101.43			1.0	Hg(II)

Determination of Analytical Greenness Metric (AGREE)

For the determination of AGREE metric, we started considering the principal aim of the analytical process that is the determination of Hg(II) ions after filtration by our AgNPs based filters. We considered the following factors:

- Parameter 1 : external treatment of the polluted water by Hg(II), with reduced number of steps.
- Parameter 2: 10 ml of polluted water and 0.4 g of filter was used for each sigle sample.
- Parameter 3: we used ICP-OES that is an off-line instrument for detecting Hg(II) ions in polluted water,.
- Parameter 4: the filtering process consists in 4 steps: filter synthesis, washing filters, Hg(II) ions solution filtering, IPC-OES measurements.
- Parameter 5: the process manual and not miniaturized.
- Parameter 6: No derivation agents are involved.
- Parameter 7: the total amount of waste is 40.4 g considering the filter (0.4 g), the sample (10 ml) and the washing process of the filter (30 ml)
- Parameter 8: the ICP-OES in the specific case, is able to detect 1 analyte with samples/hours equal to 20.
- Paramater 9: the most consuming step is the ICP-OES measurements (0.1 – 1.5 kWh per session).
- Parameter 10: none of the reagents are from bio-based sources.
- Parameter 11: Does the method involve the use of toxic reagents or solvents? YES, in total 0.1 g among $AgNO_3$ and $NaBH_4$.
- Parameter 12: Toxic to acquatic life, highly flammable and corrosive reagents are not avoided for the synthesis of AgNPs.

The final score is 0.42 as showed in the Figure S5.

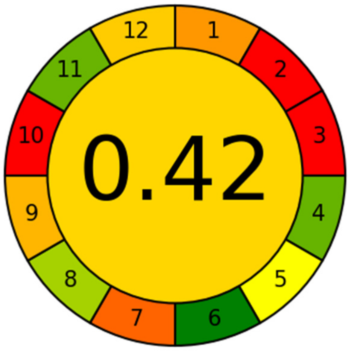

Figure S5. Result of AGREE analysis for our analytical process.

ICP-OES analysis

Commercial standard solutions of Hg and Ag (supplied by Sigma Aldrich) were used for the calibration curve of the ICP analysis. Liquid samples are introduced into plasma by peristaltic pump and discharge as an aerosol suspended in argon gas. The aerosol is carried through the centre tube of the three quartz tubes including the torch assembly in the radial plasma compartment. Sample introduction flow was fixed at 1 mL/min, while the nebulizer gas flow was fixed at 0.7 mL/min. The plasma source is powered by a 40 MHz free running solid state RF generator, the RF power was fixed at 1500 W. The plasma flux was set at 10 litre per minute (lpm); auxiliary gas flow was fixed at 0.2 Lpm. Calibration curve was constructed by leastsquare linear regression analysis of a five-point calibration curve in the range 1 – 20 ppm for Hg and 0.5 – 10 ppm for Ag. The correlation coefficient (R^2) of the calibration curve was 0.999879 for Hg and 0.999726 for Ag. The analysis wavelength for Hg was 253.625 nm, while the one used for Ag was 328.068 nm.

Calculation of the yield of polymerization

To estimate the yield of the polymerization reaction, first the the photopolymerizable solution were prepared, checking the weight of the PEGDA monomer (1.5377 g, C_i), then different filters were synthesized according to the procedure in the main text. The filters synthesis was repeated until the photopolymerizable solution was completely finished. The filters were rinsed with deionized water and dried in an oven at 120 °C for 24 h for allowing the complete evaporation of the water. After, the filters where weighted on a balance, and the final mass was 1.3865 g (C_f). From these data, we calculated the yield of the reaction according the formula $100\% - \frac{C_i - C_f}{C_i} * 100$, obtaning a value of about 90%.

Table S2. Comparison of filtering capabilities toward heavy metal ions of various nanocomposite hydrogels with gold or silver metal nanostructures.

Filtering system	Metal ion	Interaction time	Concentration range	Maximum adsorption capacity	Reference
Filter paper/AuNPs	Hg(II)	2 min	2 g/L	99% (removal efficiency)	[68]
Chitosan/AgNPs hydrogel	Cr(VI)	35 min.	0.1 μg/L – 1 mg/L	419.0 mg/g	[69]
PEGDA/AgNPs-3MPS	Hg(II)	24 h.	2 – 20 mg/L	0.58 mg/g	[70]
Nanocellulose/AuNCs*	Hg(II)	240 min.	10 – 120 mg/L	95.7 mg/g	[71]
Composed hydrogel/AgNPs	Cu(II)	2 h.	10 – 200 mg/L	111 mg/g	[72]
Composed hydrogel/AgNPs	Pb(II)	2 h.	10 – 200 mg/L	130 mg/g	[72]
3D PEGDA/AgNPs-cit-Lcys	Hg(II)	8 h.	2 – 20 mg/L	0.57 mg/g at 25°C	This work

*gold nanoclusters (AuNCs).
